# Clinical, functional, and radiological outcomes of robotic assisted versus conventional total hip arthroplasty: a systematic review and meta-analysis of randomized controlled trials

**DOI:** 10.1007/s11701-024-01949-z

**Published:** 2024-06-18

**Authors:** Pakpoom Ruangsomboon, Onlak Ruangsomboon, Khabab Osman, Daniel Pincus, Raman Mundi, Sebastian Tomescu, Bheeshma Ravi, Harman Chaudhry

**Affiliations:** 1 Division of Orthopaedic Surgery, Sunnybrook Health Sciences Centre, Toronto, ON Canada; 2 Faculty of Medicine, Siriraj Hospital, Mahidol University, Bangkok, Thailand; 3 Institute of Health Policy, Management and Evaluation, University of Toronto, Toronto, ON Canada; 4 Upstream Lab, Li Ka Shing Knowledge Institute, MAP Centre for Urban Health Solutions, St. Michael’s Hospital, Unity Health Toronto, Toronto, ON Canada; 5 Division of Orthopaedic Surgery, Department of Surgery, University of Toronto, Toronto, ON Canada; 6 ICES, Toronto, ON Canada

**Keywords:** Robotic, RATHA, Hip, Arthroplasty, Systematic review, Meta-analysis

## Abstract

This systematic review of randomized controlled trials (RCTs) aims to compare important clinical, functional, and radiological outcomes between robotic-assisted total hip arthroplasty (RATHA) and conventional total hip arthroplasty (COTHA) in patients with hip osteoarthritis. We identified published RCTs comparing RATHA with COTHA in Ovid MEDLINE, EMBASE, Scopus, and Cochrane Library. Two reviewers independently performed study screening, risk of bias assessment and data extraction. Main outcomes were major complications, revision, patient-reported outcome measures (PROMs), and radiological outcomes. We included 8 RCTs involving 1014 patients and 977 hips. There was no difference in major complication rate (Relative Risk (RR) 0.78; 95% Confidence Interval (CI) 0.22 to 2.74), revision rate (RR 1.33; 95%CI 0.08 to 22.74), and PROMs (standardized mean difference 0.01; 95%CI − 0.27 to 0.30) between RATHA and COTHA. RATHA resulted in little to no effects on femoral stem alignment (mean difference (MD) − 0.57 degree; 95%CI − 1.16 to 0.03) but yielded overall lower leg length discrepancy (MD − 4.04 mm; 95%CI − 7.08 to − 1.0) compared to COTHA. Most combined estimates had low certainty of evidence mainly due to risk of bias, inconsistency, and imprecision. Based on the current evidence, there is no important difference in clinical and functional outcomes between RATHA and COTHA. The trivial higher radiological accuracy was also unlikely to be clinically meaningful. Regardless, more robust evidence is needed to improve the quality and strength of the current evidence.

**PROSPERO registration:** the protocol was registered in the PROSPERO database (CRD42023453294). All methods were carried out in accordance with relevant guidelines and regulations.

## Background

Hip osteoarthritis is a major global contributors to lower limb disability, generally causing more severe limitations compared to knee osteoarthritis [1]. The age-adjusted incidence rate of hip osteoarthritis rose from 17.02 to 18.70 per 100,000 individuals between 1990 and 2019, suggesting an increasing burden of the condition [2].

Conventional total hip arthroplasty (COTHA) remains the primary treatment option for those unresponsive to conservative treatment. COTHA has demonstrated satisfactory longevity, with almost 60% of hip replacements lasting for 25 years [3]. With over a million procedures performed annually worldwide, optimizing surgical outcomes is essential [4]. There are potential complications that may occur, such as intraoperative fracture caused by femoral preparation with hand rasping, hip instability and limb length discrepancy (LLD) caused by improper offset restoration [5, 6]. These complications can be reduced when performed by experienced surgeons with appropriate surgical techniques [7, 8]. However, technology may also assist with the procedure and prevent such adverse consequences.

Robotic-assisted total hip arthroplasty (RATHA) was introduced in 1986 with the goal of improving implant selection, positioning, and the accuracy of bone cavity preparation [9]. It employs navigation technology to assist pre-operative planning, thus providing more precise placement and orientation of both the acetabular and femoral components than COTHA. RATHA has shown to offer more accurate implant placement, reduce the risk of dislocation in the posterior approach, and minimize LLD compared to COTHA [10], but possibly at the expense of extended operative time and more muscle damage [11].

Over the last decade, the technology of RATHA has progressed substantially. Many new advanced robotic models and software have been specifically designed for hip arthroplasty. Nevertheless, the effect of RATHA on patient outcomes is still not well understood. Also, despite multiple randomized controlled trials (RCTs) comparing RATHA with COTHA published, no systematic review and meta-analysis of these RCTs existed. Therefore, this systematic review of RCTs aims to summarize the highest quality evidence to assess whether RATHA could improve clinical, functional, and radiological outcomes compared to COTHA among patients with hip osteoarthritis.

## Materials and methods

### Study inclusion criteria

We followed the PRISMA guideline in conducting and reporting this review [12]. The protocol was registered in PROSPERO (CRD42023453294). RCTs comparing RATHA to COTHA in adult patients with hip osteoarthritis were included. We excluded studies investigating patients with high-grade hip dysplasia and studies evaluating non-RATHA technology. Studies that did not directly compare RATHA to COTHA or involving cadaveric investigations were also excluded. Only studies that evaluated important clinical, functional, and radiological outcomes defined a priori as the outcomes for this review were included in the qualitative and quantitative analysis.

### Review outcomes

The main review outcomes were (1) major complications (a composite of dislocation, loosening, and periprosthetic fracture); (2) revision rate; (3) patient-reported outcome measures (PROMs) that contain pain, walking and daily function; and (4) radiological outcomes assessed with femoral coronal stem alignment and LLD. These two radiological outcomes were chosen as the main review outcomes because of their direct relevance to postoperative function and patient satisfaction based on previous evidence [13–15]. Additional review outcomes were intra-operative blood loss, operative time, femoral stem coronal alignment outliers and radiolucency. For functional outcomes, we prespecified medium-term PROMs (at 1–5 years post-operation) as the main outcome and short-term PROMs (at 3 months post-operation) as an additional outcome.

### Database search methods

We searched Ovid MEDLINE (1946 to 2023 August 28), EMBASE (1974 to 2023 August 28), Scopus (1966 to 2023 August 28), and Cochrane Library (1908 to 2023 August 28) for eligible studies (search terms in Appendix). For the search to be sensitive, no restrictions were applied except for studies conducted in humans and in English.

### Selection of studies and data extraction

Two reviewers (P.R. and K.O.) independently reviewed the titles and abstracts of retrieved studies after duplicates were removed. Full texts were independently reviewed to confirm the eligibility of included studies, with discordances resolved through discussion. The two reviewers then independently abstracted the study data. The discordance at this stage was adjudicated by a senior reviewer (H.C.). We extracted from each study the baseline study characteristics, including the funding sources. We also recorded the study design, setting, population, interventions, and results for the outcomes of interest.

### Assessment of risk of bias

Two reviewers (O.R. and P.R.) independently assessed study-level risk of bias based on the Cochrane Collaboration’s tool for assessing risk of bias in randomized trials [16] and outcome-level risk of bias using the Risk of Bias (RoB) 2.0 tool [17]. They resolved their discordance through consensus.

### Measurement of treatment effect

We employed the random-effects models to generate the summary estimates and the generic inverse variance method to estimate the study weights. Continuous outcomes of the same metrics were analyzed and reported as mean difference (MD) and 95% confidence intervals (CIs). Functional outcomes or PROMs of different metrics, but all including components of pain, walking, and daily function, were combined and reported as standardized mean difference (SMD) [18]. We further performed a sensitivity analysis including only studies reporting the most common PROMs and analyzed the result as MD. We analyzed all categorical outcomes and presented them as risk ratio (RR) and 95%CI. Risk differences were also calculated and reported for the main outcomes to facilitate interpretation. For each review outcome analysis, we selected from each study timepoint nearest to the others to be included. For different studies from the same author evaluating the same study population at different timepoints, we only chose the most relevant timepoint from one study for each review outcome analysis to avoid sample duplication. For studies that only reported the mean values without standard deviations (SD), we used the weighted average of variances observed in other studies to calculate the SD for that study [19]. RevMan 5.4.1 (Cochrane Collaboration, Oxford) software was used to analyze all the data.

We assessed heterogeneity of the pooled analyses by visually inspecting the forest plots and exploring the *I*^2^ statistics and the Chi-squared test [20]. We further explored sources of substantial or considerable heterogeneity (*I*^2^ > 50%) with sensitivity and/or subgroup analyses as appropriate. Two sensitivity analyses were performed where available as defined a priori: (1) excluding studies with ‘high risk of bias’ and (2) excluding studies that did not employ commonly used robotic systems. We also performed two a priori subgroup analyses: (1) subgroups based on the study year of recruitment (before 2018 versus after 2018) as robotic technology has advanced significantly during the past 5 years potentially affecting the study outcomes and (2) subgroups based on the type of stem used (fully coated versus non-fully coated) as they may deliver different outcomes.

### Quality of the evidence

Two reviewers (O.R. and P.R.) independently judged the quality of evidence for each main review outcome based on the five GRADE domains [17]*.* They provided judgement on the clinical implications of the pooled effect estimates based on minimal important differences (MIDs). For complications and revision, an effect size of ≥ 0.2 was considered clinically important by consensus among experienced hip surgeons within the study team. For outcomes reported as SMD, 0.5 SD represents a moderate difference and should be considered clinically meaningful [18]. MIDs derived from the literature were at least 1 cm for LLD [15, 21] and 5° for femoral coronal stem alignment [22]. Disagreements in the grading of evidence certainty were resolved through discussion.

## Results

### Results of the search and characteristics of included studies

**Fig. 1:**
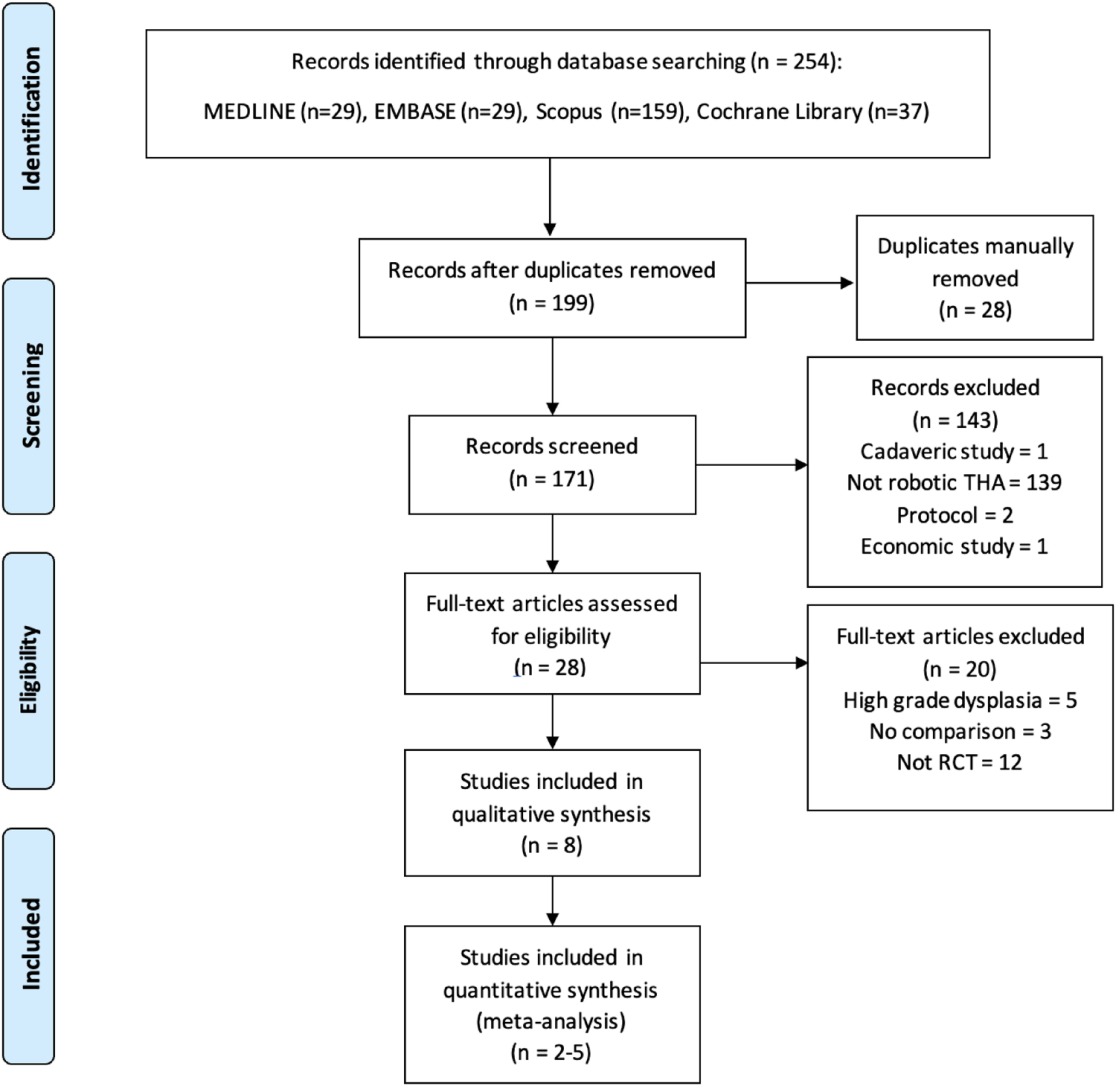
The PRISMA flow chart of study selection and inclusion. *THA* total hip arthroplasty, *RCT* randomized controlled trial

We retrieved 254 citations from the four databases; of these, 199 remained after removal of duplicates. A total of 28 full texts were evaluated for eligibility, among which 8 RCTs were included in the qualitative analysis of this review [9, 11, 23–28] (Fig. 1).

Table 1 demonstrates the characteristics of the included trials. We included 8 RCTs published over 25 years from 1998 to 2023. They were conducted in Asia (*n* = 5) [24–28], the United States (*n* = 2) [9, 23], and Germany (*n* = 1) [11], with sample sizes ranging from 54 to 154 hips and mean follow-up time between 1 month and 14 years. Two studies [23, 26] were secondary analyses of the same patient populations at longer follow-up timepoints [9, 25]. Participants’ mean age across the studies ranged from 51.2 to 71.5 years. The ROBODOC system (Integrated Surgical Systems or Curexo Technology Corp., CA, USA) was employed in most studies (*n* = 7), except one that utilized the TRex-RS (Longwell Company, Shanghai, China), the only system that could operate on both the acetabular and femoral components [28]. The VerSys fiber metal taper stem was used in three studies [25–27]. The AML or Osteoloc stem was utilized in two [9, 23] and the S-ROM in one study [11], while two other studies did not specify the stem type used [24, 28]. The posterolateral approach was the most common (*n* = 5) [9, 23, 25–27], followed by the anterolateral approach (*n* = 1) [11], and modified Hardinge approach (*n* = 1) [28], with one study not detailing the approach employed [24]. The Harris Hip Score (HHS) was the most common PROMs reported in six studies [9, 11, 23, 24, 26, 28]. Other PROMs were the Short Form-36 (SF36), the Merle d’Aubigne score, and the Western Ontario McMaster Osteoarthritis Index (WOMAC) score, which were reported in two [9, 24], three [11, 25, 27], and one study [24], respectively. Three studies declared funding sources and potential conflicts of interest [9, 24, 25].

**Table 1:** Characteristics of included trials

Author, year	Study setting	Population	Robot system	Approach, position	Acetabular cup details	Femoral stem details, head size, bearing	Follow-up time	Outcomes	Conflict of interest
Bargar 1998	United States, 3 centers, 2–4 surgeons	*N* = 120, Hips = 134 RATHA: *n* = 69, COTHA: *n* = 65, mean age – not mentioned	ROBODOC (Integrated Surgical Systems Inc, Sacramento, CA) with MIDAS Rex (Dallas, TX) milled inside of the femur	Posterior approach, lateral decubitus position	Cup model—no data Conventional technique, press-fit, screws as needed	AML (DePuy, Inc, Warsaw, IN) Hydroxyl apatite-Osteoloc (Howmedica, Rutherford, NJ) Head size—no data Bearing—no data	24 months	PROMs: HHS, SF36 Surgical related variables: operative time, blood loss, length of stay Radiographic results: femoral implant positioning Complications	None declared
Honl 2003	Germany, 1 center, 2 surgeons	*N* = 154, Hips = 141 RATHA: *n* = 61, mean age 71.5 y COTHA: *n* = 80, mean age 70.7 y	ROBODOC (Integrated Surgical Systems, Davis, California) with ORTHODOC planning computer	Anterolateral approach, lateral decubitus position	Spherical cementless (ESKA implants, Luebeck, Germany). Conventional technique, press-fit	S-ROM (DePuy, Leeds, United Kingdom) Head size—28 mm cobalt chromium Bearing—PE liner	24 months	PROMs: Merle d’Aubigne, HHS, Mayo score Surgical related variables: operative time Radiographic results: femoral implant positioning, LLD, heterotopic ossification, radiolucency Complications, revision	None declared
Nishihara 2006	Japan, 2 centers, surgeons—not mentioned	*N* = 140, Hips = 156 RATHA: *n* = 78, mean age 58 y COTHA: *n* = 78, mean age 58 y	ROBODOC (Integrated Surgical Systems, Davis, California) with ORTHODOC planning computer	Posterolateral approach, lateral decubitus position	Cup model—no data Conventional technique	VerSys fiber metal taper hydroxyapatite coated (Zimmer, Warsaw, Ind) Head size—no data Bearing—no data	Average 2.3 years (range 2–3.6 years)	PROMs: Merle d’Aubigne Physical performance test: 6 blocks walk Surgical related variables: operative time, blood loss, blood transfusion Radiographic results: femoral implant positioning, radiolucency Complications	None declared
Nakamura 2010	Japan, 1 center, 5 surgeons	*N* = 143, Hips = 162 RATHA: *n* = 81, mean age 57 y COTHA: *n* = 81, mean age 58 y	ROBODOC (Integrated Surgical Systems, Davis, California) with ORTHODOC planning computer	Posterolateral approach, lateral decubitus position	Cup model—Trilogy (Zimmer) Conventional technique	VerSys fiber metal taper hydroxyapatite coated (Zimmer, Warsaw, Ind) Head size—26 mm Bearing—Highly crosslinked PE liner (Longevity; Zimmer)	5 years	PROMs: JOA score Surgical related variables: operative time Radiographic results: radiolucency, LLD, proximal stress shielding Complications, revision	Two authors received funding from Imatron Japan Inc
Lim 2015	Sourth Korea, 1 center, 1 surgeon	*N* = 54, Hips = 49 RATHA: *n* = 24, mean age 51.2 y COTHA: *n* = 251, mean age 45.6 y	ROBODOC with ORTHODOC (Curexo Technology Corp., Fremont, CA)	No details on surgical approach, lateral decubitus position	Cup model—no data Conventional technique	Tri-lock Bone Preservation Stem (DePuy/J&J, Warsaw, IN)	24 months	PROMs: HHS, WOMAC score Surgical related variables: operative time, blood loss Radiographic results: femoral implant positioning, LLD, radiolucency Complications, revision	One author received funding from Curexo Technology
^a^Bargar 2018 (Follow-up study from Bargar 1998, combined with a new cohort)	United States, 1 center, 1 surgeon	First RCT cohort *N* = 119, Hips = 136 RATHA: *n* = 21, COTHA: *n* = 20 Second RCT cohort *N* = 97, Hips = 97 RATHA: *n* = 19,COTHA: *n* = 65	ROBODOC (Integrated Surgical Systems Inc, Sacramento, CA) with MIDAS Rex (Dallas, TX) milled inside of the femur	Posterior approach, lateral decubitus position	Cup model—no data Conventional technique, press fit, screws as needed	First RCT cohort AML (DePuy, Inc, Warsaw, IN) Hydroxyl apatite-Osteoloc (Howmedica, Rutherford, NJ) Second RCT cohort VerSys fiber metal taper hydroxyapatite coated (Zimmer, Warsaw, Ind) Hydroxyl apatite-Osteoloc (Howmedica, Rutherford, NJ) Head size—no data Bearing—no data	13.8 years for RATHA group 14.2 years for COTHA group	PROMs: HHS, SF36, WOMAC, VAS, HSQ, UCLA Complications, revision	One author disclosed potential conflict of interest
^b^Nakamura 2018 (Follow-up study from Nakamura 2010)	Japan, 1 center, 5 surgeons	*N* = 115, Hips = 128 RATHA: *n* = 64, mean age 57 y COTHA: *n* = 64, mean age 57 y	ROBODOC (Integrated Surgical Systems, Davis, California) with ORTHODOC planning computer	Posterolateral approach, lateral decubitus position	Cup model – Trilogy (Zimmer) Conventional technique	VerSys fiber metal taper hydroxyapatite coated (Zimmer, Warsaw, Ind) Head size—26 mm Cobalt-chrome and zirconia Bearing—highly crosslinked PE liner (Longevity; Zimmer)	11.25 years	PROMs: JOA score Radiographic results: radiolucency, LLD, proximal stress shielding Complications, revision	None declared
Wang 2023	China, 1 center, 2 surgeons	*N* = 72, Hips = 71 RATHA: *n* = 35, mean age 57.2 y COTHA: *n* = 36, mean age 56.3 y	TRex-RS (Longwell Company, Shanghai, China), saw blade and reamer attached to robotic arm	Modified Hardinge approach, lateral decubitus position	Cup model—no data Robot ream cup in RATHA group	Stem model—no data Robot opened canal with box osteotome, reamed femoral cavity, and implanted the femoral prosthesis	1 months	Surgical related variables: operative time Radiographic results: acetabular component related parameters, femoral implant positioning, offset restoration	None declared

*RATHA*, robotic-assisted total hip arthroplasty, *COTHA* conventional total hip arthroplasty, *PROMs* patient-reported outcome measures, *HHS* Harris Hip Score, *SF36* Short Form-36, *WOMAC* The Western Ontario and McMaster Universities Arthritis Index, *LLD* leg length discrepancy, *JOA* Japanese Orthopaedic Association, *VAS* Visual Analog Scale, *HSQ Health Status Questionnaire*, *UCLA* UCLA Activity Scale (UCLA) from University of California, Los Angeles

^a^Secondary analysis of Bargar, 1998

^b^Secondary analysis of Nakamura, 2010

### Risk of bias in included studies

**Fig. 2:**
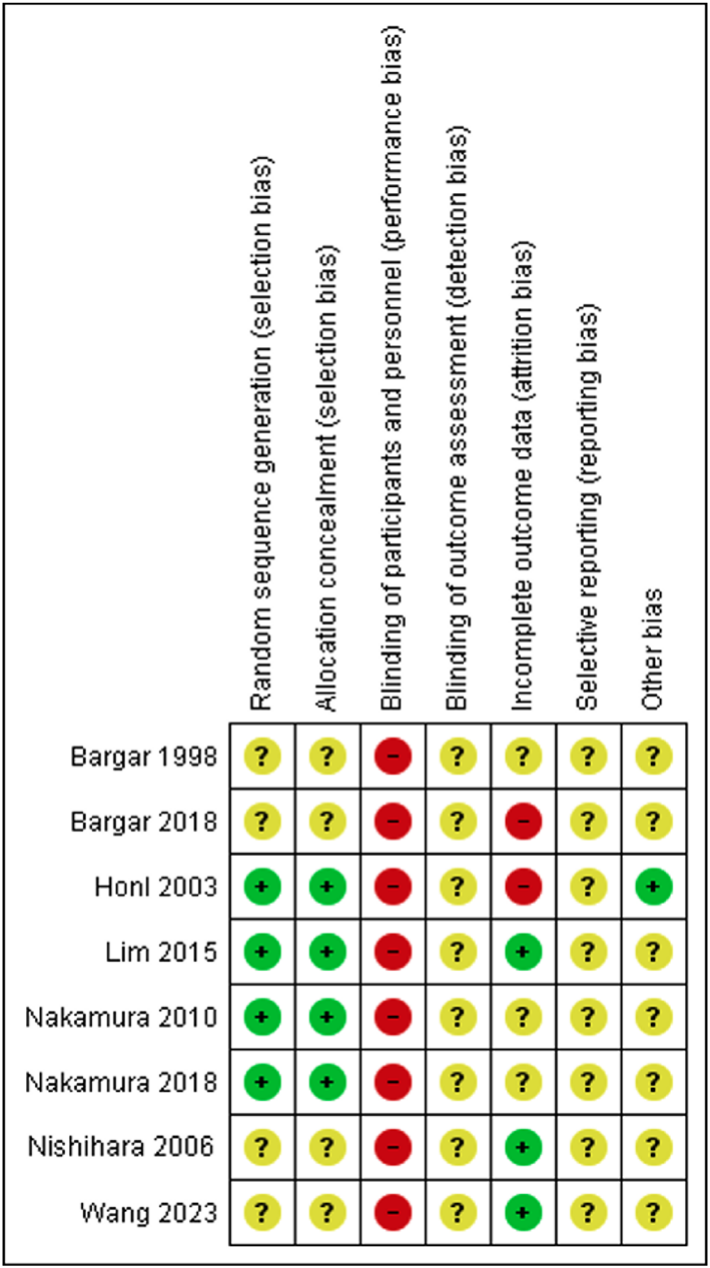
Study-level risk of bias summary

Figure 2 illustrates our judgement on the study-level risk of bias, and Table﻿ 3 elaborates the justifications for our decisions. All studies had high risk of performance bias since surgeons could not be blinded to the intervention. Most studies did not report if some outcome assessors were blinded, whether they registered their trials, nor explain how their sample size was arrived at. The outcome-level risk of bias is reported in Table 2 and the forest plot for that outcome.

**Table 2:** Risk of bias of included trials (outcome-level)

Study		Domain 1: Randomization process	Domain 2: Deviation from intervention	Domain 3: Missing outcome data	Domain 4: Measurement of outcome	Domain 5: Selective reporting	Overall risk of bias per outcome
Bargar 1998	Complication, operative time, blood loss	Some concerns^a^	Low	Low	Low	Low	**Some concerns**
	Radiographic results PROM (SF36)	Some concerns^a^ Some concerns^a^	Low Low	Some concerns^b^ Some concerns^b^	Low Some concerns^c^	Some concerns^d^ Some concerns^d^	**Some concerns** **Some concerns**
Honl 2003	Complication, operative time	Low	Low	High^e^	Low	Low	**High**
	Radiographic results	Low	Low	High^e^	Some concerns^c^	Some concerns^d^	**High**
	PROM (Harris)	Low	Low	High^e^	Some concerns^c^	Some concerns^d^	**High**
Nishihara 2006	Complication, operative time, blood loss	Some concerns^a^	Low	Low	Low	Low	**Some concerns**
	Radiographic results	Some concerns^a^	Low	Low	Low	Some concerns^d^	**Some concerns**
	PROM (Merle d’Aubigné)	Some concerns^a^	Low	Low	Some concerns^c^	Some concerns^d^	**Some concerns**
Nakamura 2010	Operative time	Low	Low	Some concerns^b^	Low	Low	**Some concerns**
	Radiographic results	Low	Low	Some concerns^b^	Low	Some concerns^d^	**Some concerns**
	PROM (JOA)	Low	Low	Some concerns^b^	Some concerns^c^	Some concerns^d^	**Some concerns**
Lim 2015	Complication, operative time, blood loss	Low	Low	Low	Low	Low	**Low**
	Radiographic results	Low	Low	Low	Low	Some concerns^d^	**Some concerns**
	PROM (Harris)	Low	Low	Low	Some concerns^c^	Some concerns^d^	**Some concerns**
Bargar 2018	Complication	Some concerns^a^	Low	High^f^	Low	Low	**High**
	Radiographic results	Some concerns^a^	Low	High^f^	Low	Some concerns^d^	**High**
	PROM (Harris)	Some concerns^a^	Low	High^f^	Some concerns^c^	Some concerns^d^	**High**
Nakamura 2018	Complication	Low	Low	Some concerns^b^	Low	Low	**Some concerns**
	Radiographic results	Low	Low	Some concerns^b^	Low	Some concerns^d^	**Some concerns**
	PROM (JOA)	Low	Low	Some concerns^b^	Some concerns^c^	Some concerns^d^	**Some concerns**
Wang 2023	Operative time	Some concerns^a^	Low	Low	Low	Low	**Some concerns**
	Radiographic results	Some concerns^a^	Low	Low	Some concerns^c^	Some concerns^d^	**Some concerns**

*PROM* patient-reported outcome measures, *JOA* Japanese Orthopedic Association

^a^No information provided on how the sequence was generated and no information on the allocation concealment method

^b^High rate (> 10%) of missingness that could have depended on the true value and affected the effect estimate

^c^Patient-reported outcome or outcomes assessed by assessors to which no information if the intervention was blinded

^d^No protocol available and the outcome can be subject to multiple analyses and selective reporting

^e^High (> 10%) and unequal (robot > conventional) rate of missingness that could have depended on the true value and affected the effect estimate

^f^Very high rate (20–40%) of missingness that could have depended on the true value and affected the effect estimate

**Table 3:** Risk of bias of included trials (study-level) and justifications for judgements

Study	Random sequence generation (selection bias)	Allocation concealment (selection bias)	Blinding of participants and personnel (performance bias)	Blinding of outcome assessment (detection bias)	Incomplete outcome data (attrition bias)	Selective reporting (reporting bias)	Other bias
Bargar 1998	Unclear No information provided on how the sequence was generated	Unclear Allocation concealment method not provided	High The intervention cannot be blinded to the surgeon(s) performing the intervention	Unclear Although radiological outcome assessors were blinded but assessors for some other outcomes were not blinded which may pose risk of bias	Unclear Some outcomes had relatively high rate of loss to follow-up	Unclear No protocol or study registration available although all outcomes stated in the methods section were reported	Unclear No information on how the sample size was arrived at
Honl 2003	Low	Low	High The intervention cannot be blinded to the surgeon(s) performing the intervention	Unclear No information whether outcome assessors were blinded	High High and unequal rate of missing outcome (RATHA > COTHA)	Unclear No protocol or study registration available although all outcomes stated in the methods section were reported	Low
Nishihara 2006	Unclear No information provided on how the sequence was generated	Unclear Allocation concealment method not provided	High The intervention cannot be blinded to the surgeon(s) performing the intervention	Unclear Although radiological outcome assessors were blinded but assessors for some other outcomes were not blinded which may pose risk of bias	Low	Unclear No protocol or study registration available although all outcomes stated in the methods section were reported	Unclear No information on how the sample size was arrived at
Nakamura 2010	Low	Low	High The intervention cannot be blinded to the surgeon(s) performing the intervention	Unclear Although radiological outcome assessors were blinded but assessors for some other outcomes were not blinded which may pose risk of bias	Unclear Relatively high rate of loss to follow-up	Unclear No protocol or study registration available although all outcomes stated in the methods section were reported	Unclear No information on how the sample size was arrived at
Lim 2015	Low	Low	High The intervention cannot be blinded to the surgeon(s) performing the intervention	Unclear Although radiological outcome assessors were blinded but assessors for some other outcomes were not blinded which may pose risk of bias	Low	Unclear No protocol or study registration available although all outcomes stated in the methods section were reported	Unclear No information on how the sample size was arrived at, potential financial conflict of interest
Bargar 2018	Unclear No information provided on how the sequence was generated	Unclear Allocation concealment method not provided	High The intervention cannot be blinded to the surgeon(s) performing the intervention	Unclear Although radiological outcome assessors were blinded but assessors for some other outcomes were not blinded which may pose risk of bias	High High rate of missing outcome data	Unclear No protocol or study registration available although all outcomes stated in the methods section were reported	Unclear No information on how the sample size was arrived at
Nakamura 2018	Low	Low	High The intervention cannot be blinded to the surgeon(s) performing the intervention	Unclear Although radiological outcome assessors were blinded but assessors for some other outcomes were not blinded which may pose risk of bias	Unclear Relatively high rate of loss to follow-up	Unclear No protocol or study registration available although all outcomes stated in the methods section were reported	Unclear No information on how the sample size was arrived at
Wang 2023	Unclear No information provided on how the sequence was generated	Unclear Allocation concealment method not provided	High The intervention cannot be blinded to the surgeon(s) performing the intervention	Unclear No information whether outcome assessors were blinded	Low	Unclear No protocol or study registration available (registration available but cannot be traced)	Unclear No information on how the sample size was arrived at

### Effects of interventions

The summary of findings for the main outcomes with the certainty of evidence is presented in the “summary of findings” table, with the reasons for rating down the evidence further elaborated in Table 4.
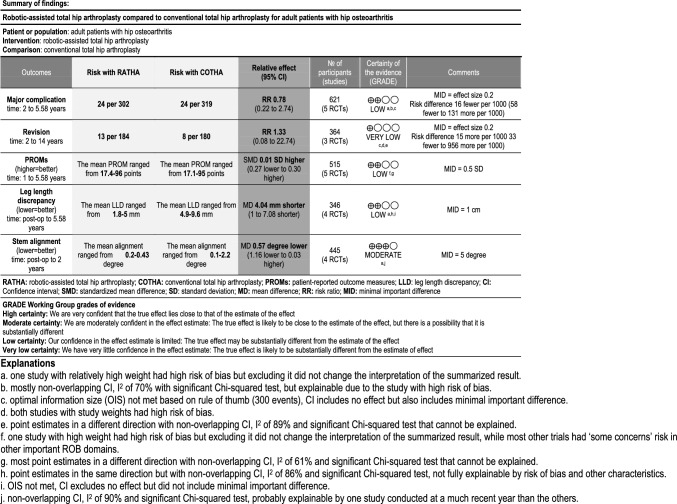


#### Major complications and revision

**Fig. 3:**
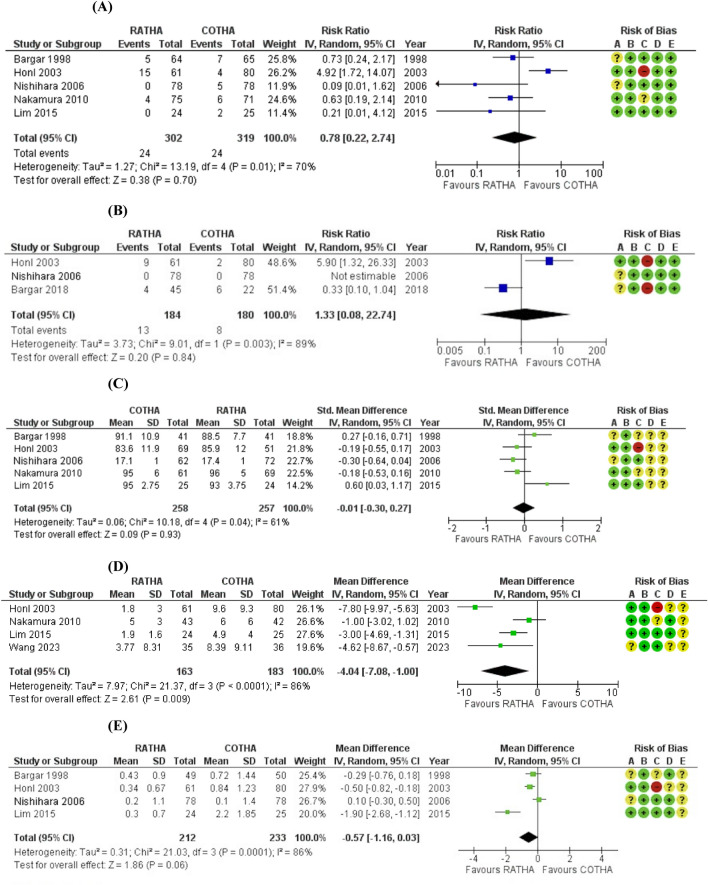
Forest plots showing major complication rate (**A**), revision rate (**B**), patient-reported outcome measures at 1–5 years (**C**), leg length discrepancy (**D**), and femoral stem coronal alignment (**E**) between robotic-assisted total hip arthroplasty (RATHA) and conventional total hip arthroplasty (COTHA). Note: outcome-level risk of bias legend; (**A**) randomization process, (**B**) deviation from intended interventions, (**C**) missing outcome data, (**D**) measurement of outcome, and (**E**) selective outcome reporting. *SD* standard deviation, *IV* inverse variance method, *CI* confidence interval, *RATHA* robotic-assisted total hip arthroplasty, *COTHA* conventional total hip arthroplasty

Five trials involving 621 hips reported major complications [9, 11, 24, 25, 27]. The combined effect showed that there was no clinically important difference between RATHA and COTHA in the rate of major complication (RR 0.78; 95%CI 0.22 to 2.74, low certainty) as shown in Fig. 3A. Although based on the point estimate, the risk of major complication with RATHA was on average 0.78 times the risk with COTHA, it only translates to a risk difference of 16 fewer per 1000. Also, the estimate was highly imprecise, with the 95%CI covering both large benefit and significant harm. Other than imprecision, the quality of evidence was low due to risk of bias, considerable inconsistency (*I*^2^ = 70%), and inadequate information size. The sensitivity analysis found all inconsistency removed (*I*^2^ = 0%), and the point estimate and 95%CI shifted further towards the benefit of RATHA (RR 0.55; 95%CI 0.26 to 1.17), but the CI still covered no effect (Fig. 5). There was no significant subgroup effect based on the stem type for major complications.

Revision rate was reported in three studies with 364 hips [11, 23, 27]. They were combined with considerable inconsistency (*I*^2^ = 89%) and with the point estimate showing a 33% higher risk for RATHA (RR 1.33) which translates to a risk increase of 15 per 1000 compared to COTHA (Fig. 3B). The combined estimate was even more imprecise (95%CI 0.08 to 22.74) than that of complications, and the majority of the combined studies had ‘high risk of bias’, both leading to the very low certainty of evidence for this outcome. Moreover, no sensitivity or subgroup analyses could be performed due to the small number of studies.

#### PROMs including pain, walking and daily function

**Fig. 4:**
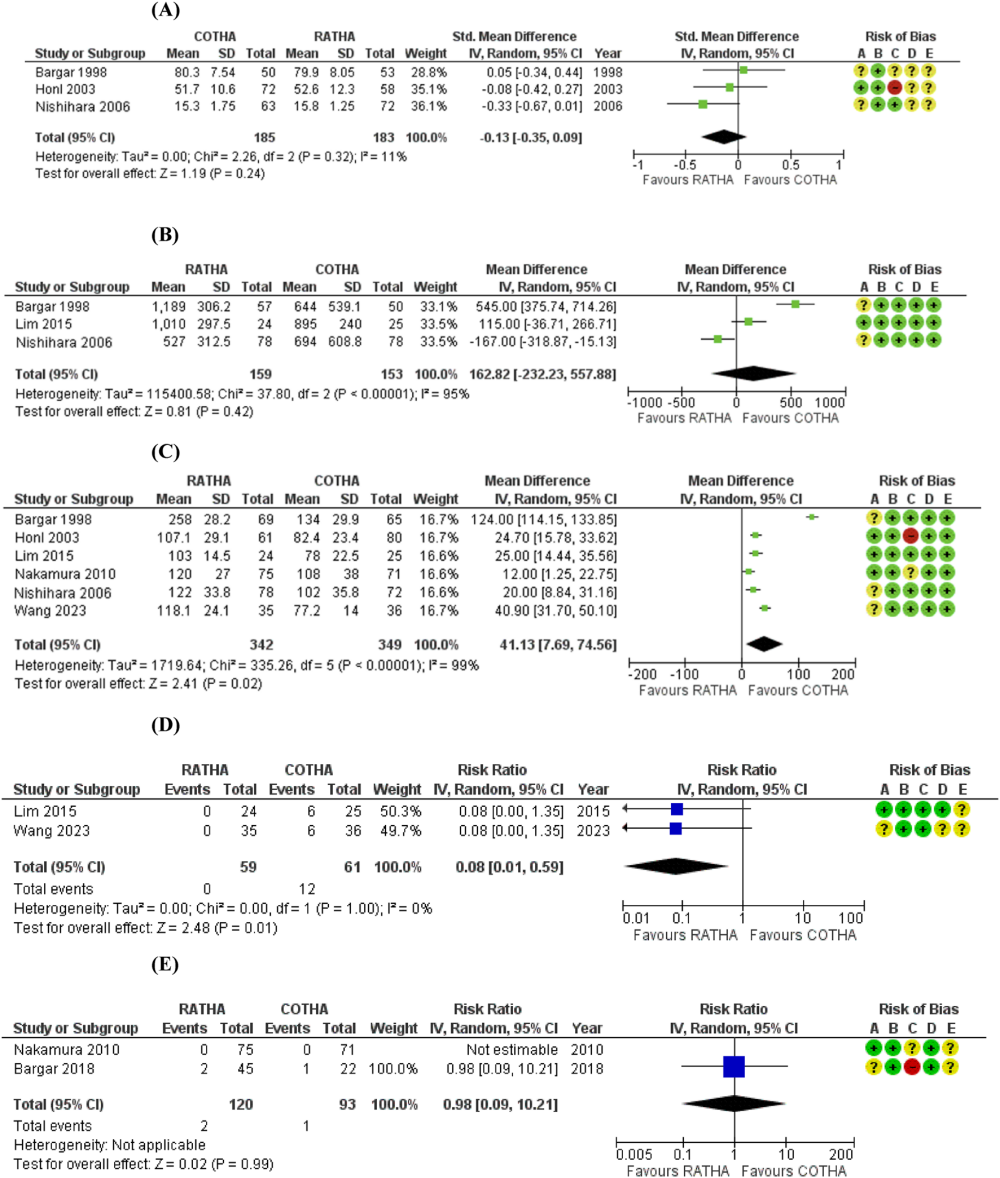
Forest plots showing patient-reported outcome measures at 3 months (**A**), intraoperative blood loss (**B**), operative time (**C**), femoral stem coronal alignment outliers (**D**), and femoral stem radiolucency (**E**) between robotic-assisted total hip arthroplasty (RATHA) and conventional total hip arthroplasty (COTHA). Note: outcome-level risk of bias legend; (**A**) randomization process, (**B**) deviation from intended interventions, (**C**) missing outcome data, (**D**) measurement of outcome, and (**E**) selective outcome reporting. *SD* standard deviation, *IV* inverse variance method, *CI* confidence interval, *RATHA* robotic-assisted total hip arthroplasty, *COTHA* conventional total hip arthroplasty

**Fig. 5:**
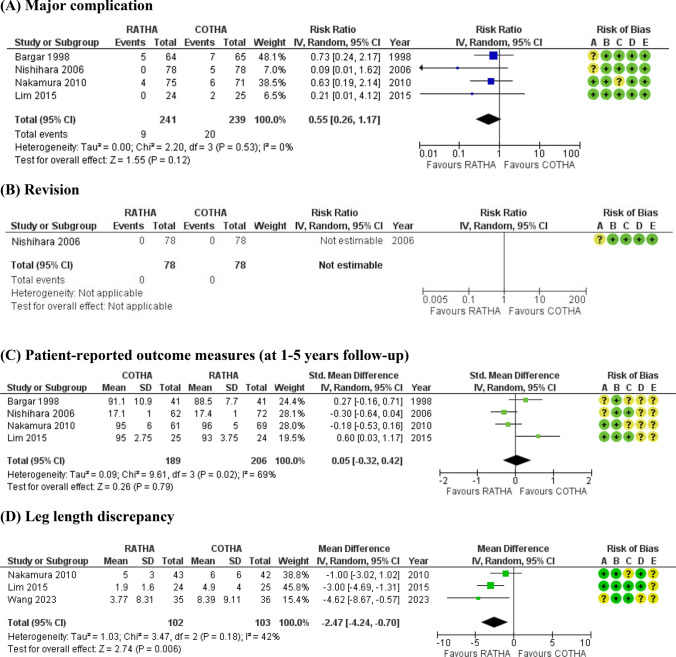

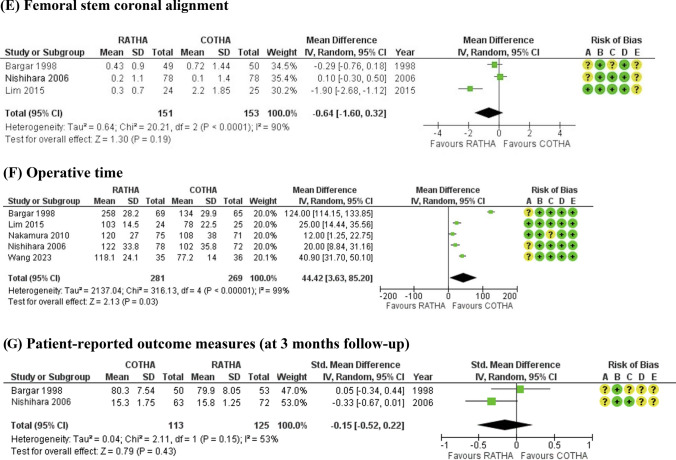
Forest plots showing major complication rate (**A**), revision rate (**B**), patient-reported outcome measures at 1–5 years (**C**), leg length discrepancy (**D**), femoral stem coronal alignment (**E**), operative time (**F**), and patient-reported outcome measures at 3 months (**G**) between robotic-assisted total hip arthroplasty (RATHA) and conventional total hip arthroplasty (COTHA) excluding studies with high risk of bias. Note: outcome-level risk of bias legend; (**A**) randomization process, (**B**) deviation from intended interventions, (**C**) missing outcome data, (**D**) measurement of outcome, and (**E**) selective outcome reporting. *SD* standard deviation, *IV* inverse variance method, *CI* confidence interval, *RATHA* robotic-assisted total hip arthroplasty, *COTHA* conventional total hip arthroplasty

**Fig. 6:**
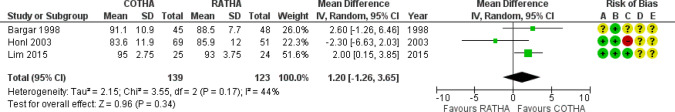
Forest plot showing Harris Hip Score at 24 months between robotic-assisted total hip arthroplasty (RATHA) and conventional total hip arthroplasty (COTHA). Note: outcome-level risk of bias legend; (**A**) randomization process, (**B**) deviation from intended interventions, (**C**) missing outcome data, (**D**) measurement of outcome, and (**E**) selective outcome reporting. *SD* standard deviation, *IV* inverse variance method, *CI* confidence interval, *RATHA* robotic-assisted total hip arthroplasty, *COTHA* conventional total hip arthroplasty

Medium-term PROMs were evaluated in five studies involving 515 patients [9, 11, 24, 25, 27], three of which reported the HHS [9, 11, 24]. RATHA resulted in almost no difference in PROMs compared to COTHA (SMD 0.01; 95%CI − 0.27 to 0.30 SD, low certainty) as presented in Fig. 3C. There was substantial inconsistency (*I*^2^ = 61%) that cannot be explained by risk of bias or other study characteristics. The sensitivity analyses of studies without ‘high risk of bias’ (Fig. 5) and with only the HHS (Fig. 6) yielded similar results with very small between-group differences. Similarly, short-term PROMs reported in 3 studies of 368 patients [9, 11, 27] favored RATHA based on the point estimates (0.12 SD higher for RATHA), but the effect size was not likely clinically important, and the 95%CI covered no effect (-0.09 to 0.35 SD) as shown in Fig. 4A. No subgroup effect based on the stem type was found for both PROMs outcomes.

#### Radiological outcomes

**Fig. 7:**
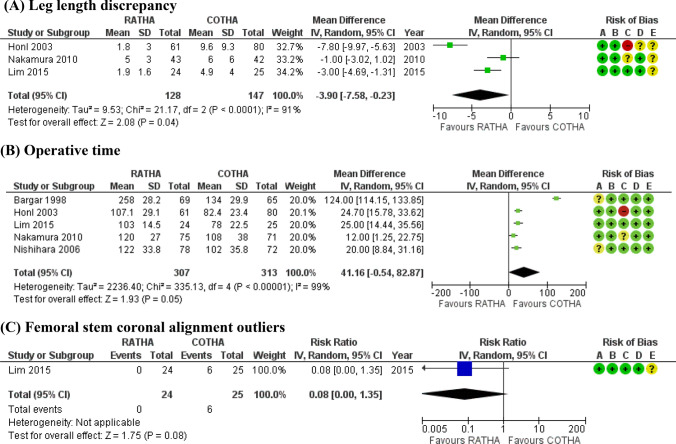
Forest plots showing leg length discrepancy (**A**), operative time (**B**), and femoral stem coronal alignment outliers (**C**) between robotic-assisted total hip arthroplasty (RATHA) and conventional total hip arthroplasty (COTHA) including only studies that employed commonly used robotic systems. Note: outcome-level risk of bias legend; (**A**) randomization process, (**B**) deviation from intended interventions, (**C**) missing outcome data, (**D**) measurement of outcome, and (**E**) selective outcome reporting. *SD* standard deviation, *IV* inverse variance method, *CI* confidence interval, *RATHA* robotic-assisted total hip arthroplasty, *COTHA* conventional total hip arthroplasty

**Fig. 8:**
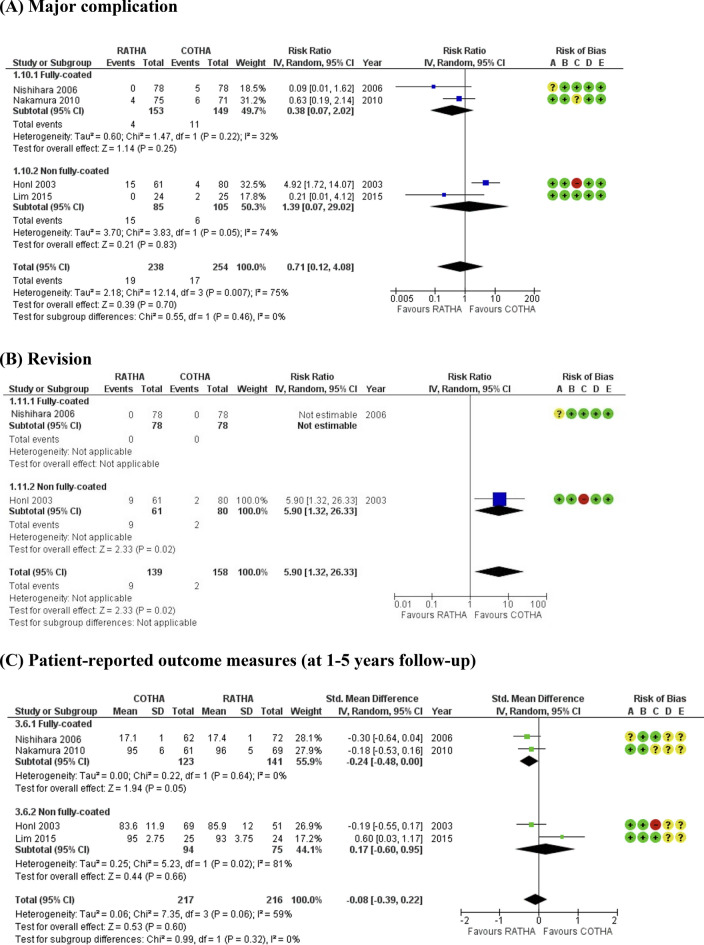

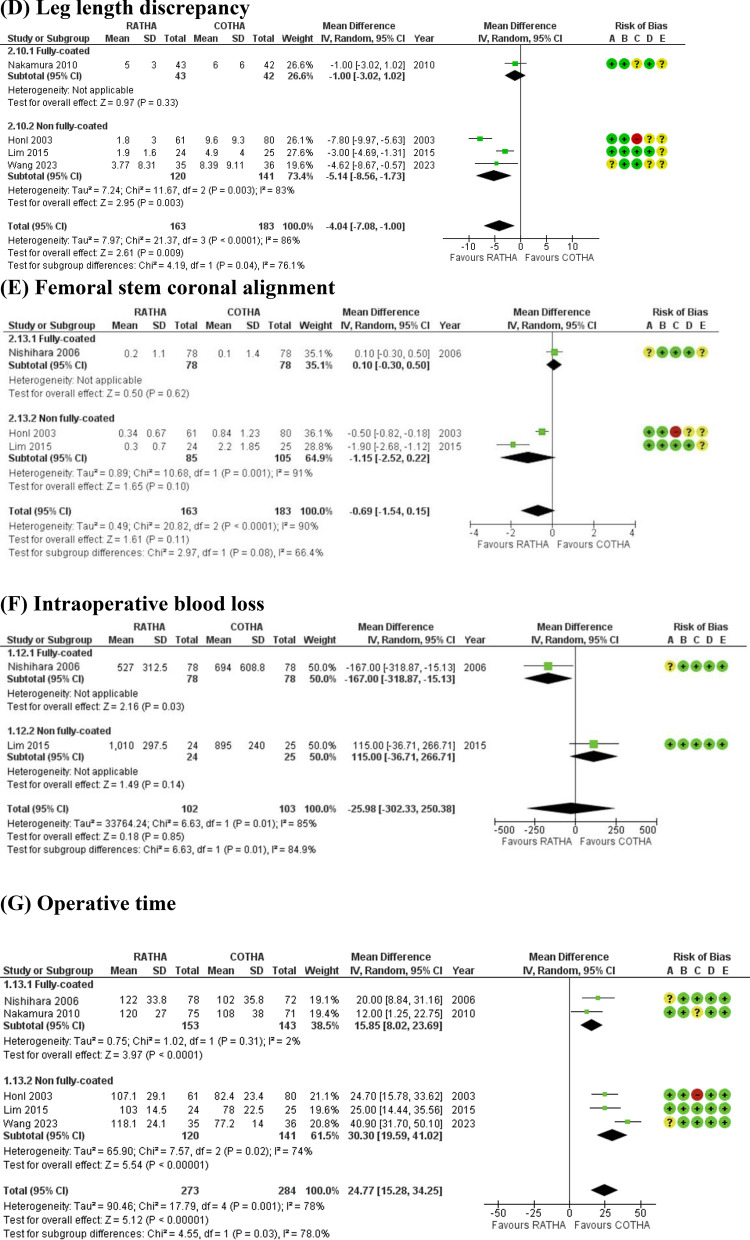

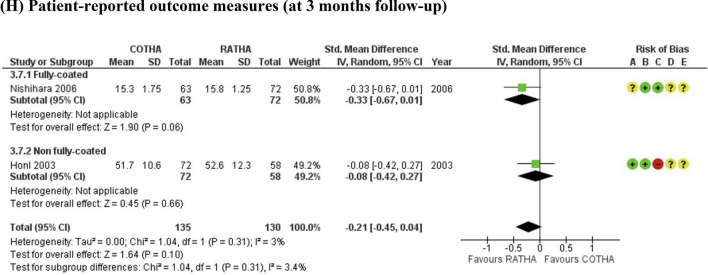
Forest plots showing subgroup analyses based on type of stem used for major complication rate (**A**), revision rate (**B**), patient-reported outcome measures at 1–5 years (**C**), leg length discrepancy (**D**), femoral stem coronal alignment (**E**), intraoperative blood loss (**F**), operative time (**G**), and patient-reported outcome measures at 3 months (**H**) between robotic-assisted total hip arthroplasty (RATHA) and conventional total hip arthroplasty (COTHA). Note: outcome-level risk of bias legend; (**A**) randomization process, (**B**) deviation from intended interventions, (**C**) missing outcome data, (**D**) measurement of outcome, and (**E**) selective outcome reporting. *SD* standard deviation, *IV* inverse variance method, *CI* confidence interval, *RATHA* robotic-assisted total hip arthroplasty, *COTHA* conventional total hip arthroplasty

**Fig. 9:**
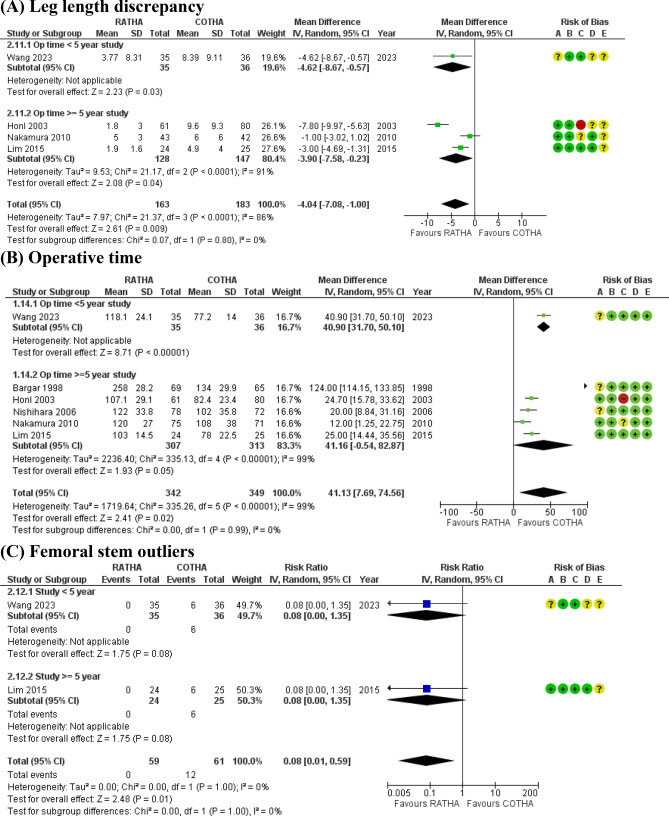
Forest plots showing subgroup analyses based on study recruitment year for leg length discrepancy (**A**), operative time (**B**), and femoral stem outliers (**C**) between robotic-assisted total hip arthroplasty (RATHA) and conventional total hip arthroplasty (COTHA). Note: outcome-level risk of bias legend; (**A**) randomization process, (**B**) deviation from intended interventions, (**C**) missing outcome data, (**D**) measurement of outcome, and (**E**) selective outcome reporting. *SD* standard deviation, *IV* inverse variance method, *CI* confidence interval, *RATHA* robotic-assisted total hip arthroplasty, *COTHA* conventional total hip arthroplasty

For LLD, the results from four studies [11, 24, 25, 28] recruiting 346 hips were combined with considerable heterogeneity (*I*^2^ = 86%). LLD for RATHA was on average 4 mm less than that of the COTHA group (95%CI − 7.08 to − 1.0 mm, low certainty) (Fig. 3D). Both sensitivity analyses found results in the same direction favoring RATHA but still with at least substantial inconsistency and small effect sizes that are not clinically meaningful (Figs. 5 and 7). Studies that used non-fully coated stem yielded significantly lower LLD for RATHA compared to those employing fully coated stem (*p*-value = 0.04) (Fig. 8), though no subgroup effect was found based on study recruitment year (Fig. 9).

Pooled results of 445 hips from 4 studies [9, 11, 24, 27] showed that RATHA resulted in a trivial reduction in femoral coronal stem alignment than COTHA (effect size of 0.57°), with 95% CI including no effect (− 1.16° to 0.03°, moderate certainty) (Fig. 3E). The sensitivity analysis excluding ‘high risk of bias’ studies gave concordant result (Fig. 5). There were no significant subgroup effects, although a tendency towards better alignment in favor of RATHA was found in studies using non-fully coated stems (Fig. 8).

#### Additional outcomes

Overall, RATHA resulted in higher intraoperative blood loss (MD 162.8 ml; 95%CI − 232.2 to 557.9 ml) and prolonged operative time (MD 41.1 min; 95%CI 7.7 to 74.6 min) compared to COTHA (Fig. 4B, C). The superiority of COTHA over RATHA for these two outcomes was significantly more prominent if non-fully coated stem was used (Fig. 8). However, RATHA yielded significantly lower rate of femoral stem coronal alignment outliers (RR 0.08; 95%CI 0.01 to 0.59) (Fig. 4D), although it was not superior to COTHA in terms of preventing femoral stem radiolucency (RR 0.98; 95%CI 0.09 to 10.21) (Fig. 4E). Regardless, it is important to note that the very low number of pooled participants and events in the analyses of these two radiologic measurements.

## Discussion

Based on the highest quality current evidence, this systematic review and meta-analysis of RCTs suggests that there is little to no difference in clinical, functional, and radiological outcomes between RATHA and COTHA. The effect sizes for major complications and revision rate were relatively small and accompanied by very large 95%CIs. Our pooled estimate also showed almost negligible difference in PROMs between the two surgical modalities. Although the pooled estimates on LLD and femoral coronal stem alignment tended towards RATHA delivering better component positioning, the effect sizes were largely smaller than MID and unlikely to be clinically meaningful.

Robotic-assisted surgery has recently been introduced and gained significant attention as an innovative surgical option in hip and knee arthroplasty. Although it may provide better surgical accuracy, there is limited high-quality evidence on its effects on important clinical outcomes. A recent systematic review and meta-analysis of RCTs found that robotic-assisted total knee arthroplasty (RATKA) is not superior to conventional techniques in terms of functional outcomes, and the evidence on complications and revision rate is still lacking despite its increasing popularity worldwide [29]. As for RATHA, an earlier systematic review in 2019, which included a mixture of RCTs and observational studies, reported comparable functional outcomes between RATHA and COTHA [30]. Similarly, a recent network meta-analysis revealed no significant differences in revision and complication rates between the two surgical options [31]. Our results pooled from more up-to-date and higher quality of evidence were concordant with these previous reviews. Therefore, based on the current best evidence, both RATKA and RATHA have yet to demonstrate superior clinical efficacy compared to conventional modalities. This may imply that important clinical outcomes may not largely depend on the technology but still rely very much on surgical experience and techniques.

Nevertheless, although we could not demonstrate an important difference in major complications between RATHA and COTHA as the primary analysis found largely imprecise estimate and a small effect size, we discovered a large effect size in favor of RATHA and the 95%CI almost excluding no effect in the sensitivity analysis without ‘high risk of bias’ studies. All inconsistency also disappeared, thus supporting potential evidence certainty. This finding suggests that statistically significant and clinically important superiority of RATHA over COTHA in terms of major complication could have possibly been demonstrated with more high-quality evidence or larger RCTs with low risk of bias. These future studies are required to ascertain this potential benefit of RATHA over COTHA.

However, there was inadequate data to achieve high-quality evidence regarding revision rate. With such a small number of studies and participants in this outcome analysis, the combined estimate was at very low certainty level; thus, no clinical implications should be made. The lack of evidence for this outcome could have been because of the long lifespan of hip replacements. Many RCTs collecting this data are ongoing and following up on patients’ outcomes, as evidenced by several trial registrations we found upon performing the search for this review. Interestingly, most of these ongoing RCTs employ newer robotic systems, some of which have haptic-feedback features that limit safety boundaries for instrumenting, and can operate both acetabular and femoral sides. These advanced features may lead to better clinical outcomes for RATHA.

In terms of functional outcomes, we found no important difference in medium-term and short-term PROMs between RATHA and COTHA. Our findings are consistent with an earlier systematic review of RCTs and observational studies that revealed no significant differences in PROMs, pain, quality of life, and satisfaction, regardless of the timeframe considered [30]. Our primary analysis only found a trivial effect size of 0.01 SMD in favor of RATHA for medium-term PROMs. Since this result in SMD unit may be difficult to comprehend, we further performed a sensitivity analysis for HHS, the most common PROMs among all. Although its point estimate favored COTHA, it was very small compared to the whole scale (1.2 points from a score ranging from 0 to 100 points) and not considered clinically important [32, 33]. Also, the 95%CI covered no effect and did not include the MID, thus supporting the conclusion that there is no important difference in PROMs between the two surgical procedures. Regardless, we found that short-term PROMs was more inclined to favor RATHA than medium-term PROMs, contrasting with the findings of the previous review [30]. This discrepancy could have been attributed to the inclusion of Bargar et al.’s study in our analysis, which was not considered in their review. Nevertheless, it is essential to note that the certainty of evidence is at low level due to risk of bias and unexplained inconsistency. Therefore, more methodologically robust studies are required to confirm this result and strengthen our conclusion.

Even though RATHA has shown to improve radiological outcomes in terms of higher rates of component placement within the safe zones [34, 35], we could not demonstrate such findings. Although LLD was overall more precise for RATHA, the effect size was too small to be clinically meaningful [15]. Moreover, we found little to no difference in femoral coronal stem alignment between the two groups, with a trivial pooled effect size, 95% CI covering no effect and excluding an important difference, and a moderate certainty level of evidence. One possible reason behind the negative effects in terms of radiological outcomes was that most included trials employed the ROBODOC system, which is optimized for the femoral side rather than acetabular component orientation, overall biomechanical alignment, and offset restoration. Consequently, our analysis reflects the limitations of this robotic technology, which may not capture the full spectrum of potential radiological benefits and may be inferior to other robotic systems that can address and prepare for both femoral and acetabular components.

Nevertheless, despite insignificant and unimportant differences in the primary analyses of radiological outcomes, there were notable subgroup effects favoring RATHA if non-fully coated stems were used. This could have been because of the milling precision of RATHA that facilitates an optimal placement of non-fully coated stems due to their lower frictional resistance, allowing for more precise implantation to the desired position compared to their fully coated counterparts. Regardless, it is important to note that these subgroup analyses were based on a small number of studies, especially in the fully coated stem subgroup, where only one study was analyzed. Similar issues were found in the analyses for femoral stem outliers and radiolucency. Only one or two studies with scarce events were combined for these analyses, thus making the pooled estimates imprecise and of very low certainty. Consequently, these analyses were likely underpowered, and future studies are needed to confirm their findings.

Additionally, we found that RATHA yielded relatively more blood loss and longer operative time, both of which were worse if non-fully coated stems were used. The subgroup difference could have been due to the more meticulous femoral preparation and extensive bone milling required for non-fully coated stems to achieve an optimal fit [24], as compared to the straight fully coated stems, which permit more straightforward and swifter milling [25, 27]. Furthermore, we initially expected to find lower blood loss and shorter operative time for RATHA initiated after 2018, though we did not see such a finding for operative time and could not perform the subgroup analysis for intraoperative blood loss due to the lack of studies recruiting patients after 2018. This issue of inadequate number of studies also raised concerns when interpreting the pooled estimates as previously discussed.

This review had limitations. Most of the included trials had moderate-to-high risk of bias, and in some of the analyses, there were only a small number of included trials and participants, both of which lowered the certainty of the evidence. The latter also caused imprecise and potentially biased consistency and treatment effect estimates. Funnel plots could not be created to assess reporting bias due to the same reason. Furthermore, we faced the scarcity of recent trials, with only one trial commencing after 2018. A plausible explanation could be the disruption caused by the COVID-19 pandemic, which necessitated the reallocation of healthcare resources away from elective surgery. An additional limitation arises from the fact that the robotic system employed in seven of the eight studies is ROBODOC, which has been phased out by several countries due to its limited clinical benefits. As previously mentioned, many new trials have just begun with modern robot system after the pandemic eased and are still ongoing and awaiting medium-to-long-term patient follow-up, thus not yet published. These newer trials employing more advanced robotic technologies that may yield better outcomes for RATHA can limit the generalizability of our findings; this review should thus be replicated once they are published. In addition, we could not evaluate some important outcomes of hip arthroplasty, such as the accuracy of acetabular component placement, because they were not comprehensively reported. The inclusion of future RCTs employing up-to-date robotic technology assessing all relevant outcome domains with robust methodology and adequate information size can potentially lead to higher certainty in the evidence and more definitive conclusions.

## Conclusion

Based on the current evidence, there is no clinically important difference in clinical, functional, and radiological outcomes between RATHA and COTHA, as we found no significant and meaningful difference in terms of major complication and revision rates, PROMs that evaluate pain, walking and daily function, and femoral coronal stem alignment. Although RATHA results in significantly lower LLD than RATHA, the difference is not clinically meaningful. Future well-designed and rigorously conducted RCTs evaluating up-to-date robotic systems and focusing on all important outcomes are required to improve the quality and strength of the current evidence.

## Data Availability

No datasets were generated or analysed during the current study.

## Appendix: Search strategies

Ovid MEDLINE: Epub Ahead of Print, In-Process & Other Non-Indexed Citations, Ovid MEDLINE® Daily and Ovid MEDLINE® < 1946-August 2023 > 

Date of search: 28 August 2023.

**Table:**

#	Search
1	Osteoarthritis, Hip/
2	(Osteoarthritis Hip or OA Hip or Hip arthrosis patient or osteoarthritic Hip or Coxarthrosis or Primary Coxarthrosis).tw,kf
3	1 or 2
4	Robotic Surgical Procedures/
5	Robotics/
6	(Robo or Robot* or RATHA or RATHR or Robotic arm* or assisted Robotic device* or Robotic surgical device* or Robotic-aided surger*).tw,kf
7	4 or 5 or 6
8	Arthroplasty, Replacement, Hip/
9	((conventional or traditional or primary or total) adj3 (hip replacement or hip arthroplasty or hip surgery or hip resur-fac*)).tw,kf
10	8 or 9
11	3 and 7 and 10

EMBASE: Embase Classic + Embase < 1947 to 2023 August > 

Date of search: 29 August 2023.

**Table:**

#	Search
1	exp hip osteoarthritis/
2	(Osteoarthritis hip or OA hip or hip arthrosis patient or osteoarthritic hip or Coxarthrosis or Primary coxarthrosis).tw,kf
3	1 or 2
4	robot assisted surgery/
5	exp robotics/
6	exp robot/
7	(Robo or Robot* or Robotic or Robotic surgery or RATHA or RATHR or Robotic arm* or newly designed robotic arm or active robotic-assisted total hip or robotic milling or Robodoc or assisted Robotic device* or Robotic surgical device* or Robotic-aided surger*).tw,kf
8	4 or 5 or 6 or 7
9	total hip arthroplasty/ or total hip prosthesis/
10	exp hip replacement/
11	((conventional or traditional or total or primary) adj3 (hip replacement or hip arthroplasty or hip surgery or hip resur-fac*)).tw,kf
12	9 or 10 or 11
13	3 and 8 and 12

SCOPUS < 1966 to 2022 August 23 > 

Date of search: 28 August 2023.

**Table:**

#	Search
1	TITLE-ABS-KEY ( hip AND osteoarthritis)
2	(TITLE-ABS-KEY (hip AND osteoarthritis)) OR (SUBJAREA (medi OR nurs OR vete OR dent OR heal OR mult) TITLE-ABS-KEY ((hip AND osteoarthritis))) OR (TITLE-ABS-KEY ((hip AND osteoarthritis))) AND (LIMIT-TO (SRCTYPE, "j"))
3	TITLE-ABS-KEY ((hip AND osteoarthritis)) AND (LIMIT-TO (SRCTYPE, "j"))
4	(TITLE-ABS-KEY ((hip AND osteoarthritis))) OR (TITLE-ABS-KEY ((hip AND osteoarthritis))) OR ((TITLE-ABS-KEY (hip AND osteoarthritis)) OR (SUBJAREA (medi OR nurs OR vete OR dent OR heal OR mult) TITLE-ABS-KEY ((hip AND osteoarthritis))) OR (TITLE-ABS-KEY ((hip AND osteoarthritis)))) AND (LIMIT-TO (SRCTYPE, "j"))
5	TITLE-ABS-KEY (robot AND assisted AND surgery)
6	TITLE-ABS-KEY (robotics)
7	TITLE-ABS-KEY (robot)
8	(TITLE-ABS-KEY (robot AND assisted AND surgery)) OR (TITLE-ABS-KEY (robotics)) OR (TITLE-ABS-KEY (robot)) OR (TITLE-ABS-KEY (robo OR robot* OR robotic OR robotic AND surgery OR ratka OR ratkr OR robotic AND arm* OR assisted AND robotic AND device* OR robotic AND surgical AND device* OR robotic-aided AND surger* OR robo* AND resurfac*))
9	TITLE-ABS-KEY (total AND hip AND arthroplasty/OR total AND hip AND prosthesis/)
10	TITLE-ABS-KEY (hip AND replacement)
11	TITLE-ABS-KEY (conventional OR traditional OR total OR primary AND hip AND replacement OR hip AND arthroplasty)
12	(TITLE-ABS-KEY (conventional OR traditional OR total OR primary AND hip AND replacement OR hip AND arthroplasty)) OR ((TITLE-ABS-KEY (total AND hip AND arthroplasty/OR total AND hip AND prosthesis/)) OR (TITLE-ABS-KEY (hip AND replacement)) OR (TITLE-ABS-KEY (conventional OR traditional OR total OR primary AND hip AND replacement OR hip AND arthroplasty OR resufac*)))
13	((TITLE-ABS-KEY (conventional OR traditional OR total OR primary AND hip AND replacement OR hip AND arthroplasty)) OR ((TITLE-ABS-KEY (total AND hip AND arthroplasty/OR total AND hip AND prosthesis/)) OR (TITLE-ABS-KEY (hip AND replacement)) OR (TITLE-ABS-KEY (conventional OR traditional OR total OR primary AND hip AND replace-ment OR hip AND arthroplasty OR resufac*)))) AND ((TITLE-ABS-KEY (robot AND assisted AND surgery)) OR (TITLE-ABS-KEY (robotics)) OR (TITLE-ABS-KEY (robot)) OR (TITLE-ABS-KEY (robo OR robot* OR robotic OR robotic AND surgery OR ratha OR rathr OR robotic AND arm* OR assisted AND robotic AND device* OR robotic AND surgical AND device* OR robotic-aided AND surger* OR robo* AND resurfac*))) AND ((TITLE-ABS-KEY (hip AND osteoarthritis)) OR (SUBJAREA (medi OR nurs OR vete OR dent OR heal OR mult) TITLE-ABS-KEY ((hip AND osteoarthritis))) OR (TITLE-ABS-KEY ((hip AND osteoarthritis)))) AND (LIMIT-TO (SRCTYPE, "j"))

Cochrane Library < 1908 to 2022 August 22 > 

Date of search: 28 August 2022.

**Table:**

#	Search
#1	MeSH descriptor: [Osteoarthritis, Hip]
#2	(Osteoarthritis hip or OA hip or hip arthrosis patient or osteoarthritic hip or coxarthrosis or Primary conarthrosis or avascular necrosis hip or dysplastic hip):ti,ab,kw
#3	#1 OR #2
#4	[mh ^"robot assisted surgery"]
#5	[mh "robotics"]
#6	[mh "robot"]
#7	(Robo or Robot* or Robotic or Robotic surgery or RATHA or RATHR or Robotic arm* or assisted Robotic device* or Robotic surgical device* or Robotic-aided surger*):ti,ab,kw
8	#4 or #5 or #6 or #7
9	[mh "Arthroplasty"]
10	((conventional or traditional or primary or total) NEAR/3 (hip replacement or hip arthroplasty or hip replacement or hip arthroplasty or milling or rasping or hip surgery or hip resurfac*)):ti,ab,kw
11	#9 OR #10
12	#3 AND #8 AND #11

See Tables 3 and 4.

**Table 4:** Details on the GRADE assessments of quality of evidence TO APPENDIX

Outcomes	Domain 1 Risk of bias	Domain 2 Inconsistency	Domain 3 Indirectness	Domain 4 Imprecision	Domain 5 Publication bias	Overall quality of evidence
Major complication	Serious limitations, downgrade 0.5 level Reasons: 1/5 study with the highest weight had high risk of bias, but excluding it did not change the interpretation of the summarized result	Serious limitations, downgrade 0.5 level Reasons: I^2^ of 70% with significant Chi-squared test, but explainable due to the study with high risk of bias already acknowledged in domain 1	No serious limitations not downgrade Reasons: patients, interventions, outcomes, and methodology of included studies were similar to those of this review	Serious limitations, downgrade 1 level Reasons: optimal information size (OIS) not met based on rule of thumb (300 events), CI includes no effect but also includes minimal important difference	No serious limitations not downgrade Reasons: Included studies were from a comprehensive search	Low
Revision	Serious limitations, downgrade 1 level Reasons: both studies with weights had high risk of bias	Serious limitations, downgrade 1 level Reasons: point estimates in a different direction, non-overlapping CI, I^2^ of 89% with significant Chi-squared test of unexplainable source		Serious limitations, downgrade 1 level Reasons: OIS not met, CI includes no effect but also includes minimal important difference		Very Low
PROM	Serious limitations, downgrade 0.75 level Reasons: 1/5 study had high risk of bias but excluding it did not change the interpretation of the summarized result. Most other trials had ‘some concerns’ risk in other important ROB domains	Serious limitations, downgrade 1 level Reasons: most point estimates in a different direction with non-overlapping CI and I^2^ of 61% and significant Chi-squared test that cannot be explained		No serious limitations, not downgrade Reasons: OIS met by rule of thumb (n = 400), CI covers no effect and excludes possible important benefit		Low
LLD	Serious limitations, downgrade 0.5 level Reasons: 1/5 study with 26% weight had high risk of bias, but excluding it did not change the interpretation of the summarized result	Serious limitations, downgrade 0.75 level Reasons: point estimates in the same direction but with non-overlapping CI, I^2^ of 86% and significant Chi-squared test, not fully explainable by risk of bias in domain 1		Serious limitations, downgrade 1 level Reasons: OIS not met by rule of thumb, CI excludes no effect but did not include minimal important difference		Low
Stem alignment	Serious limitations, downgrade 0.5 level Reasons: 1/4 study with 28% weight had high risk of bias but excluding it did not change the interpretation of the summarized result	Serious limitations, downgrade 0.75 level Reasons: non-overlapping CI, I^2^ of 86% and significant Chi-squared test, probably explainable by one study conducted at a much recent year than the others		No serious limitations, not downgrade Reasons: OIS met by rule of thumb, CI covers no effect and excludes possible important benefit		Moderate

See Figs. 5, 6, 7, 8 and 9.
